# The Use of Implementation Intentions to Promote Vitamin D Supplementation in Young Children

**DOI:** 10.3390/nu4101454

**Published:** 2012-10-12

**Authors:** Jascha de Nooijer, Roos Jansen, Patricia van Assema

**Affiliations:** 1 Department of Health Promotion, NUTRIM School for Nutrition, Toxicology and Metabolism, Maastricht University Medical Centre, PO Box 616, 6200 MD, Maastricht, The Netherlands; Email: p.vanassema@maastrichtuniversity.nl; 2 GGD Rotterdam-Rijnmond, Postbus 70032, 3000 LP Rotterdam, The Netherlands; Email: r.jansen3@rotterdam.nl

**Keywords:** vitamin D, supplementation behaviour, implementation intentions, intervention, young children, parents

## Abstract

Only 50% of Dutch children aged 0–4 years receive sufficient daily vitamin D supplementation. This study aims to investigate the effectiveness of implementation intentions in promoting vitamin D supplementation among young children. An electronic survey was conducted among parents of children aged 0–4 (*n* = 171). These parents were randomly assigned to two groups: one that received implementation intention instructions and one that did not. At follow-up, there were no significant between group differences in any outcome measures. These results suggest that merely asking parents to formulate an implementation intention with respect to giving their child daily vitamin D supplementation is insufficient to improve vitamin D intake among young children. However, testing the intervention via the Internet may not have allowed us to exploit the full potential of the strategy. Investigation of the use of implementation intentions in the setting of toddler consultation clinics is therefore recommended.

## 1. Introduction

Vitamin D facilitates calcium metabolism and bone mineralization, and is therefore essential in the development of healthful bones during childhood [1]. It is known to prevent rickets in children, and a vast and increasing body of scientific research suggests that it may also lower the risk of developing various other diseases at a later age, including osteoporosis, cardiovascular disease, diabetes mellitus, auto-immune diseases and certain cancers [1,2,3]. 

Vitamin D can be produced in our skin due to sunlight (ultraviolet radiation). It may also be (partly) obtained from a limited number of dietary sources, such as fatty fish, or foods fortified with vitamin D, such as margarine. However, as the daily amount of vitamin D needed in young children is much higher than that provided by synthesis in the skin or food intake, the Health Council of the Netherlands recommends providing children aged 0–4 with an additional 10 μg of vitamin D per day in the form of drops or pills. Despite these recommendations, low levels of vitamin D intake among young children in the Netherlands have been found. The mean intake for children aged 2–6 is 3.6 μg/day [1] and, although most parents have a high intention to give their child vitamin D supplementation [4], only 50% of Dutch children receive the recommended supplementation of 10 μg/day [4,5].

The formation of implementation intentions may provide a simple and effective solution to prompt parents to actually act upon their good intentions to give their child vitamin D [6,7,8]. An implementation intention is a specific “if–then” plan about where, when and how to perform a certain goal-directed behaviour (“If situation X is encountered, then I will initiate behaviour Y.”). Implementation intentions create a mental link between a specific situation and a certain desired behaviour. As a result, the goal-directed behaviour is enacted relatively effortlessly and automatically as soon as the critical situation is encountered [6,7,8,9]. This implies that implementation intentions are effective for people who are already motivated [10], and that they help to remind people to act as they intended. Implementation intentions have been found to be effective in stimulating a wide range of health-promoting behaviours, such as physical activity, fruit consumption, sunscreen use, cancer-screening attendance, medical self-examinations and taking vitamin C pills [11,12,13,14]. Sheeran and Orbell found that implementation intentions were effective in increasing vitamin C intake, but in this study, vitamin pills were provided [13]. No studies were found that used implementation intentions to promote vitamin D supplementation in young children. 

A pre-test among parents of children aged 0–4 years showed that the formation of an implementation intention helped to remind them to give their child vitamin D supplementation [15]. The present study was designed to gain further insight into the effectiveness of implementation intentions in stimulating vitamin D intake among young children. If effective, the use of implementation intentions might be a promising, but also simple and time efficient, strategy for public health nurses to encourage parents to provide their child with adequate vitamin D supplementation. In the Netherlands, parents with children up to the age of 4 regularly attend toddler consultation clinics staffed by professional medical doctors and nurses where they discuss the child’s healthy development. Vitamin D is one of the topics discussed during these visits. For practical reasons we test the effects of implementation intentions among parents of young children via an Internet panel. If effects are positive in a controlled setting, we could implement this in toddler consultation clinics, according to the framework for designing and evaluation of complex interventions [16]. We did not provide vitamin D supplements, while this will also not be done in real life situations. It was hypothesized that formation of an implementation intention, as compared to no formation of such an intention, would result in: (1) a higher number of parents performing adequate supplementation behavior; (2) a higher number of days on which parents give vitamin D supplementation to their child; (3) a higher number of parents who have vitamin D supplementation at home.

## 2. Experimental Section

### 2.1. Participants and Procedures

A randomized, controlled trial was conducted with follow-up after four weeks. The initial study sample consisted of 894 parents of children aged 1 to 3½ years old, residing in all parts of the Netherlands. The parents were registered members of Flycatcher, a Dutch ISO-certified Internet panel with data from 20,000 people. The parents who were requested to fill out a questionnaire on vitamin D and children and responded, received an incentive of €2 for each completed questionnaire. Participants were randomly allocated to either the control group (questionnaire without implementation intention intervention) or experimental group (questionnaire with implementation intention intervention). In the case of multiple children under the age of 3½, participants were instructed to fill out the questionnaires regarding their youngest child. Participants already performing adequate supplementation behaviour (defined as giving the child 10 μg of vitamin D supplementation at least six days a week), participants with a negative intention towards giving vitamin D supplementation, participants not living together with the child and, in the experimental group, participants who did not fill in an implementation intention were excluded from participation. Under Dutch regulations, no ethical approval was required for this type of study.

### 2.2. Questionnaires

The questionnaire was adapted from an earlier questionnaire measuring the determinants of vitamin D supplementation in young children in the Netherlands [4]. 

The first section consisted of questions on *child characteristics* including sex, age, first-born status, whether the child lived together with the parent and the child’s diet (e.g., breastfed, formula fed). 

*Present supplementation-related behaviour* was measured by two items on how many days in the previous week the child had received vitamin D supplementation, and whether the participant had vitamin D supplements at home. 

To eliminate differences in knowledge between participants, participants in both groups received written information on the Health Council’s advice on vitamin D supplementation, including an explanation why vitamin D is important in children and the amount of vitamin D that is required. 

*Intention to give vitamin D supplementation *was assessed with two items on a 5-point Likert scale: “Do you intend to give your child the recommended amount of vitamin D (10 μg) every day this week?” (*yes, certainly * to *no, certainly not*) and “How sure are you that you will give your child the recommended amount of vitamin D (10 μg) every day this week?” (*very sure * to * not at all sure*). A mean score for intention was computed (α = 0.80). 

Participants in the experimental condition were then asked to formulate an implementation intention towards giving their child 10 μg vitamin D per day. They were presented with the following instruction: “Many people find it hard to act on their good intentions. However, if you make a specific plan that specifies *where* and *when* you are going to do *what*, your chance of success will increase. We would like to ask you to make such a plan for giving extra vitamin D to your child. Describe the moment you want to do this. Choose a moment that you think is most suitable. It is important that you be as specific as possible.” Participants were provided with an example, after which they filled out their own implementation intention (e.g., “*If I bring my child to bed and just before reading the bedtime story, I will give my child 10 μg vitamin D.*”).

To conclude, participants’ demographic characteristics, including sex, age, occupation, education, country of origin and partner’s country of origin, were asked. 

At follow-up, present supplementation-related behaviour and intention to give vitamin D supplementation were reassessed. Participants in the experimental condition received five additional process evaluation questions concerning their implementation intention: whether they could remember the plan they had made (*yes*/*no*), whether they had executed the plan as intended (*yes*/*no*), whether they had found it difficult to make a plan (*not difficult at all * to *very difficult*), whether they had found it useful (not *useful at all *to * very useful*) and whether the plan had prompted them to start giving vitamin D to their child (*yes*/*no*). 

### 2.3. Statistical Analyses

Descriptive statistics were used to describe demographic characteristics and supplementation-related behaviour at baseline. One-way ANOVAs and chi-square tests were used to check randomization. Attrition was studied by means of logistic regression analysis with attrition as the dependent and condition, participants’ sex, age, educational level, ethnicity, intention and supplementation-related behaviour at baseline as independent variables. 

To evaluate the effect of implementation intentions on adequate vitamin D supplementation behaviour (≥6 days a week), a logistic regression analysis was conducted with adequate supplementation behaviour at follow-up as the dependent, and condition (0 = control, 1 = intervention) as the independent variable. Adequate supplementation behaviour at baseline was not included in the regression analysis since this variable was constant for all participants. The effect on the number of days that parents gave their child vitamin D supplementation was tested using a linear regression analysis, with the number of days parents gave their child vitamin D supplementation at follow-up as the dependent, and condition and number of days that parents gave their child vitamin D supplementation at baseline as independent variables. Furthermore, paired sample T-tests per group were undertaken to assess the difference in number of days between baseline and follow-up in both groups. Finally, to see whether implementation intentions had a positive effect on the number of parents who had vitamin D supplementation at home, a logistic regression analysis was conducted with vitamin D at home at follow-up as the dependent, and condition and vitamin D at home at baseline as independent variables. All analyses were conducted using SPSS version 15.0 and significance was set at *P* < 0.05. 

## 3. Results

### 3.1. Response, Dropouts and Participants

**Figure 1:**
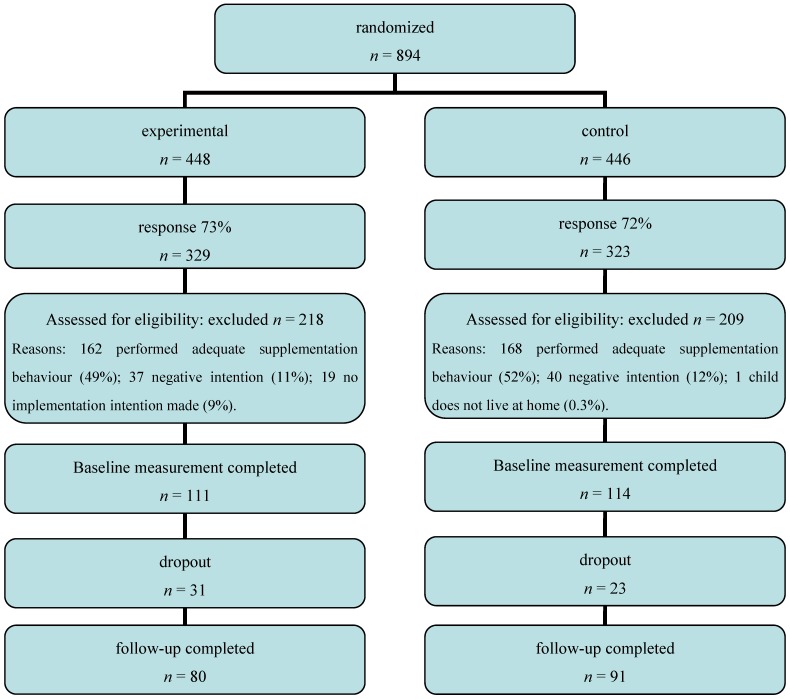
Flow of participants through the study.

The flow of participants through the study is shown in Figure 1. In total, 894 participants were approached to take part in the study of which 652 (73%) responded. The final sample consisted of 171 participants who completed both measures. Logistic analyses of dropout did not reveal selective dropout between baseline and follow-up. No differences were found between the experimental and the control condition at baseline on demographic characteristics, intention to give vitamin D, number of days participants gave vitamin D and whether participants had vitamin D at home, indicating that the randomization procedure was successful. 

The majority of the participants were female (78.4%), with a mean age of 34.1 years (SD = 4.4). Most participants (60.2%) had a high level of education, 36.2% a medium level and 3.5% a low level. Most children were of Dutch origin (90.6%) and mean age was 2.3 years (SD = 0.8) (Table 1). 

**Table 1:** Baseline characteristics of participants (*n* = 171).

Characteristic	% or Mean ± S.D.
Participant sex (female/male)	78/22
Participant age (years)	34.1 ± 4.4
Participant education level (low/med/high)	4/36/60
Child sex (girl/boy)	54/46
Child age (years)	2.3 ± 0.8
First-born child (yes/no)	40/60
Child ethnicity (autochthonous/allochthonous)	91/9
Intention to give vitamin D (0–2)	1.2 ± 0.6
Number of days child received vitamin D (1–5)	2.02 ± 2.0
Vitamin D at home (yes/no)	81/19

### 3.2. Supplementation-Related Behaviour at Baseline

Approximately 50% of the participants reported to have provided vitamin D supplementation to their child on 6 or 7 days a week, while 10% expressed a negative intention towards giving their child vitamin D supplementation and were therefore excluded from further analyses (Figure 1). Of the participants, 42% reported that they never provided their child with vitamin D, while 8% reported that they did so on 1 day, 10% on 2 days, 10% on 3 days, 10% on 4 days and 21% on 5 days a week. On average, participants gave their children vitamin D supplementation on 2 days a week. Eighty-one percent of the participants had vitamin D supplements at home. 

### 3.3. Effects of Implementation Intentions

The percentage of participants who never provided their child with vitamin D decreased from 32 (40%) to 16 participants (19%) in the experimental group and from 39 (43%) to 26 participants (29%) in the control group. At follow-up, 29 participants (36%) in the experimental group *versus* 25 (27%) in the control group had started to give their child vitamin D supplementation on at least 6 days a week (adequate supplementation behaviour) (Table 2). However, this improvement in the experimental group was not significantly different from the improvement in the control group. 

After four weeks, participants gave their children vitamin D supplements on average on 4.1 days per week in the experimental group and 3.5 days per week in the control group (Table 2). This is a significant increase of 2.1 days per week in the experimental group (t = 6.64, *P* < 0.001) and 1.5 days in the control group (t = 5.87, *P* < 0.001). However, the difference in increase in the number of days on which participants gave their children vitamin D supplementation between the experimental and the control group was not significant.

Last, the number of participants with vitamin D supplements at home increased by 8 (10%) in the experimental group, *versus* 4 (4.4%) in the control group (Table 2). This difference between the experimental and the control group was borderline significant (OR 3.54, *P* = 0.05, 95% CI 0.97–12.59), indicating that implementation intentions might have had a positive effect on the number of participants with vitamin D at home. 

**Table 2:** Outcome measures in experimental (*n* = 80) and control (*n* = 91) groups at baseline and follow-up.

	Baseline		Follow-up	
	Experimental group	Control group	Experimental group	Control group
Number of parents performing adequate supplementation behaviour	- *	- *	29 (36%)	25 (27%)
Mean number of days parents gave vitamin D supplementation to their child ± S.D.	2.0 ± 2.0	2.0 ± 2.1	4.1 ± 2.5	3.5 ± 2.7
Number of parents with vitamin D supplements at home	67 (84%)	72 (79%)	75 (94%)	76 (84%)

* Parents already performing adequate supplementation behaviour at baseline were excluded from the study.

### 3.4. Evaluation by Participants

Seventy percent (*n* = 56) of the participants in the experimental group remembered the plan they had made at baseline. Of these participants, 46% (*n* = 26) felt that formulating a plan had been useful, and 38% (*n* = 21) found it had been rather useful. More than half of these participants (*n* = 31) answered that the plan had helped them to start giving their child vitamin D supplementation. 

## 4. Discussion

The present paper studied the effects of implementation intentions to provide children (0–4 years) with 10 µg of vitamin D per day on (1) the number of parents who perform adequate supplementation behavior, (2) the number of days that the child was given vitamin D supplementation and (3) the number of parents who had vitamin D supplementation at home. 

Our results confirm earlier studies indicating that, although almost all parents have a high intention to supply their child with vitamin D, only 50% of children receive adequate supplementation. Unfortunately, we have to conclude that the implementation intentions used here did not result in improvements on any of the outcome measures at traditional levels of significance (*P* < 0.05), which contrasts with earlier findings that implementation intentions positively influenced various health behaviours [12] including vitamin C pills [13]. 

There may be several explanations for the lack of significant effects of the implementation intention intervention in this study. First, the intervention might have been too simple to be effective. We tested the effects of implementation intentions via a certified Internet panel before investigating their impact in toddler consultation clinics. This meant that parents had to form implementation intentions by themselves, and we could not control whether they took the time to think seriously about a plan or whether their plans were realistic and specific enough. Guiding participants with forming implementation intentions could have led to more realistic plans [17], which could be a facilitated at toddler consultation clinics by training nurses in this domain.

Second, the study sample (*n* = 171) could have been too small to detect differences between the groups. Although the Internet panel we used contains over 20,000 participants, there were only 900 participants in the target group. Of the participants, about 50% already performed the desired behaviour (conform [5]) or did not have a positive intention toward vitamin D supplementation. The remaining group was probably too small to get significant results of the intervention. Moreover, it is possible that the intervention took participants by surprise, as questionnaires from this Internet panel do not usually include an intervention.

A third explanation could be a test effect of filling out the questionnaire. Participants in the control group may have been influenced by the extra attention for vitamin D supplementation or prompted to remember information about vitamin D that they had received in the past, which may explain why participants in the control group showed improvements on all outcome measures as well. So, parents are cued by filling in the questionnaire, and subsequently, there is limited additional value of forming implementation intentions. However, it is possible that only when implementation intentions are formulated, an automatic behavioural response is created due to the repeated responses in the same situation. If so, possibly, long term effects may only be achieved when implementation intentions are formulated. 

The sample in our study consisted mainly of highly educated parents of Dutch origin. This does not fully represent the general Dutch population. Children of non-European parents are particularly vulnerable to vitamin D deficiency, due both to darker skin pigmentation that limits the conversion of vitamin D and (in some cultures) to the use of long sleeves and pants, and/or the use of oil instead of fortified margarine. We therefore recommend also studying the impact of implementation intentions on vitamin D supplementation among immigrant parents and parents with lower educational levels. 

This study suggests that merely asking parents to formulate a plan towards giving their child daily vitamin D supplementation is insufficient to improve vitamin D intake among young children to desired levels. As mentioned above, we suspect that by using electronic questionnaires via an Internet panel to test our intervention we have not exploited the full potential of the strategy. Given the positive evaluation by the participants and better improvements in the experimental group on all outcome measures, we believe it is worth exploring the use of implementation intentions in the setting of toddler consultation clinics in order to stimulate habitual vitamin D supplementation. If nurses are trained to discuss implementation intentions with the parents, this could lead to more specific plans and probably to greater effects on vitamin D intake. However, pre-structured implementation intentions require less training of nurses. It has been stated that implementation intentions are most effective if the critical situation is clearly described. As a consequence, it is easier to recognize the situation and deliberation when encountering the situation is no longer necessary [7,11]. Reminding participants of the plan they have made, for instance by sending a text message to their mobile phone or writing the plan on a fridge magnet, might also improve the impact of the implementation intention. It would therefore be worthwhile to investigate the willingness of child health clinics to include these simple strategies in their usual care. 

## 5. Conclusions

The current study confirms the need to improve vitamin D supplementation behaviour of parents for their children, and as implementation intentions are simple, time-efficient and inexpensive, they could well be an interesting strategy for child health clinics to stimulate parents to stick to their intentions. 
